# Tracking Children’s Physical Activity Patterns across the School Year: A Mixed-Methods Longitudinal Case Study

**DOI:** 10.3390/children7100178

**Published:** 2020-10-12

**Authors:** Irfan Khawaja, Lorayne Woodfield, Peter Collins, Adam Benkwitz, Alan Nevill

**Affiliations:** 1 Department of Sport and Exercise, Birmingham City University, Birmingham B15 3TN, UK; 2 Department of Social Science, Sport and Business, Newman University, Birmingham B32 3NT, UK; L.A.Woodfield@staff.newman.ac.uk (L.W.); A.Benkwitz@staff.newman.ac.uk (A.B.); 3 Faculty of Education, Health and Wellbeing, University of Wolverhampton, Wolverhampton WS1 3BD, UK; Peter.Collins@wlv.ac.uk (P.C.); a.m.nevill@wlv.ac.uk (A.N.)

**Keywords:** global positioning system, physical activity, location, mixed methods, longitudinal, children

## Abstract

Despite the breadth of health benefits associated with regular physical activity (PA), many children in the UK are not sufficiently active enough to meet health guidelines, and tend to become less active as they mature into and throughout adolescence. Research has indicated that children’s school, home and neighbourhood environments can all significantly influence their opportunities to engage in moderate-to-vigorous physical activity (MVPA). However, less is known about how children’s MVPA patterns within these key environments may change across the school year. The current mixed-methods case study aims to explore this issue by tracking key stage 2 (KS2) and key stage 3 (KS3) children’s MVPA patterns across the school year. Fifty-eight children (29 boys, 29 girls, KS2 = 34, KS3 = 24) wore an integrated global positioning systems (GPS) and heart rate (HR) monitor over four consecutive days in the first term of school (autumn), before these measurements were repeated in the two remaining school terms (winter–summer). A subsample of children (n = 6–8 per group) were invited to take part in one of six focus groups each term to further explore their PA behaviours and identify the barriers and facilitators to PA. The children’s MVPA was significantly lower (*p* = 0.046) in term 2 (winter/spring term) than during the warmer terms (autumn and summer). All the locations showed reductions in MVPA in term 2, except indoor MVPA, which increased, and MVPA on foot in the neighbourhood, which remained consistent. Focus groups revealed location, friends, and the variety of options to be associated with MVPA, and poor weather, parental permission, and time limitations to be barriers to MVPA. This mixed-methodological, repeated-measures design study highlights differences in the activity patterns and perceptions of children over the school year. Future studies should implement longitudinal, multi-method approaches to gain deeper insight into how children’s PA behaviours differ over time. Consequently, this can inform future health policies promoting children’s PA throughout the year.

## 1. Introduction

Research exploring children’s physical activity (PA) indicates that many children fail to meet the daily 60 min moderate-to-vigorous physical activity (MVPA) guidelines [1,2], and sedentary behaviour (SB) is highly prevalent [3]. These findings have been related to numerous environmental factors, including weather and daylight hours, which are considered to be important determinants of children’s PA and SB patterns [4], although this area of research is currently under-explored [5]. Previous evidence suggests that lower levels of children’s PA are consistently observed in winter when compared with summer [6,7], and seasonal variation in relation to PA exerts a stronger influence on children than on adolescents [8]. Additional research in Western countries indicates that higher temperatures and lower precipitation is associated with higher levels of PA [5], with one study indicating an optimal maximum temperature of 20–25 °C for children’s MVPA levels [9]. The organisational setting, PA intensity, the number of different PAs available, and the motive for participation are strongly correlated with seasonal variation [10]. For example, increased PA participation using indoor sports facilities are associated with poorer outdoor weather conditions [10].

Over the past two decades, research has taken greater interest in the impact of the built environment on health behaviours such as PA and dietary habits [11]. This research has been aided by the introduction of global positioning system (GPS) tracking devices, which, when combined with other objective measures, enable the objective measurement of children’s movement and MVPA patterns within their surrounding built environment. Such research, which combined GPS and accelerometry in measuring children’s PA across the school year, has revealed increased amounts of children’s MVPA during summer months compared with winter [12], with children spending more time in the home environment during cold winter months and spending more time outdoors on streets and in purpose-built outdoor spaces (e.g., parks and playgrounds) during the warmer spring and summer months [12]. The literature indicates that seasonality and weather conditions as determinants of PA should be explored further, particularly in reference to specific countries [13]. Moreover, research that includes objective tracking measures within a mixed-methodological approach could provide further insightful evidence to inform future health interventions. Mixed-methods approaches to exploring children’s PA produce reflective findings, where quantitative research focuses on common trends, and qualitative methods explore “how” and “why” questions [14]. Research exploring children’s PA has largely underrepresented children’s voices [15]. Additionally, research has been limited to singular qualitative methods that overlook children’s varied linguistic ability and interaction preference [15]. When researching children’s PA, both the type and context of PA is important, in addition to what PA and how much PA they do [16]. Qualitative measures including focus groups provide opportunities for children to expand on their reasons for PA behaviour, and allow the researcher to discern the meaning behind children’s PA, which would therefore support mixed-methods research design [16]. Therefore, this study will aim to address the following research questions: Does children’s PA location and intensity change across the school year? Do children’s reasons to engage/not engage in PA differ according to different times of the school year?

## 2. Materials and Methods

### 2.1. Participants

This study adopted a similar mixed-methods approach to a previous investigation [17]. A sample of children (9–13 years) from a middle school in the West Midlands, United Kingdom, was invited to take part in the study (school data: 560 total children; 17% from ethnic minority groups; 21% free school meal eligibility, which is greater than the current 13.6% national average [18]). The school’s head teacher, parents/guardians, and children provided informed consent for inclusion before the children participated in the study. The study was conducted in accordance with the Declaration of Helsinki, and the protocol was approved by the Ethics Committee of Newman University, Birmingham (2015-12-22-0500334/2395). Following the study promotion at the school, an opportunity sample of 119 children aged 9–13 years (21% of the total school population) provided consent. After cleaning the GPS and heart rate (HR) data, 58 out of the 119 children’s profiles met the one-hour minimum data inclusion criteria in term 1, as did 50 children in term 2 and 41 children in term 3, respectively. Therefore, these data sets were used for analysis (see Table 1).

### 2.2. Procedures

Children received a tutorial on how to use and wear GPS and HR equipment, including details on how to wear the heart rate monitor chest strap and operating and recharging the GPS watch. Children were also advised on when equipment should not be worn (i.e., activities which were water-based, sports which included contact, etc.). GPS watches (Garmin Forerunner 305, Garmin Ltd., Olathe, KS, USA) and synchronised HR monitors were worn daily over a four-day period, and this time period has been supported by previous studies [19]. The four-day data collection period enabled two weekdays to be compared with two weekend days (Thursday–Sunday, as soon as children woke up until going to bed), and this provided a reflection of the children’s daily PA.

Following the GPS and HR data collection periods, a random stratified sample (according to gender and key stage) of 6–8 children per group were invited to participate in a focus group each school term (18 focus groups in total). Previous literature supports the use of focus groups with children [20]. The lead researcher led the focus groups, which were facilitated by a research assistant. The focus groups took place in a classroom during school lunch time. The focus groups were recorded using an mp3 audio recorder, and the emergent themes were cross-checked with the research assistant.

Focus groups topics for discussion were underpinned by the social–ecological model, which identifies opportunities to promote PA considering the individuals characteristics (e.g., gender, beliefs, and attitudes), behaviours (sedentary and active time), social interactions (family, teachers, and peers), and physical environments (e.g., the availability of PA equipment and facilities) that influence their ability to engage in PA [21]. A deductive approach to constructing focus group questions was based upon PA and GPS data already collected. The focus groups explored the children’s reasons behind their PA behaviour and PA location. PA facilitators and barriers to PA were also explored within the focus groups.

### 2.3. Data Processing and Statistical Analysis

The Garmin Connect website (Garmin Ltd., Olathe, KS, USA) was used to download the GPS and HR data. Raw matching GPS and HR data were extracted using the GPS Utility software. Children’s locations were established by manually entering the children’s location points into Google Maps. Children’s visited locations provided a location index of: “Home”, “School”, “Other indoor location”, “Motorised transport”, “On foot”, “Outdoors”, and “Outside” (combining “on foot” and “outdoors”). Further data cleansing took place by analysing the children’s profiles and removing the missing GPA and HR time fields.

Children’s PA intensity thresholds were calculated using each child’s heart rate reserve, which was followed by applying the Karvonen method to calculate the percentage training intensities [22]. MVPA was the intensity used to explore the children’s PA within this study, and this was established by combining Moderate Physical Activity (MPA) and Vigorous Physical Activity (VPA). MPA was classified as 50%–75% of a child’s maximum HR, and any HR data above 75% of a child’s maximum heart rate was classified as VPA [23]. 

Data sets which have a hierarchical structure can be explored in order to examine interactions between the “layers” within data sets [24]—e.g., within this study, the school being one layer, the key stage being a second layer, and gender being a third layer. Location data and MVPA gender and key stage interactions were investigated using a repeated-measures analysis of variance (ANOVA) test over the three school terms. A significance value of *p* < 0.05 (two-tailed) was considered as being statistically significant for all the analyses. Descriptive statistics were presented for the analyses, which included the mean MVPA in each location and the numbers of children meeting the recommended daily PA guidelines. SPSS version 25 (SPSS Inc., Chicago, IL, USA) was used for these statistical analyses.

As with the previous study [17], a thematic analysis was used on the transcriptions to understand the reasons behind the children’s PA [14,25]. This provided insight into the reasons/motives for children’s PA each school term [26].

## 3. Results

### 3.1. Children’s MVPA Levels across the School Year

Children engaged in significantly fewer MVPA minutes in term 2, comprising of winter/spring months (F₁₁,₁₃₅ = 3.140, *p* = 0.046, Eta^2^ = 0.044), and a Bonferroni pairwise comparison revealed a statistically significant difference between terms 1 (which comprised autumn months) and term 2 (term 1 MVPA = 83.8 min, term 2 MVPA = 48.2 min; *p* = 0.028). 

**Figure 1:**
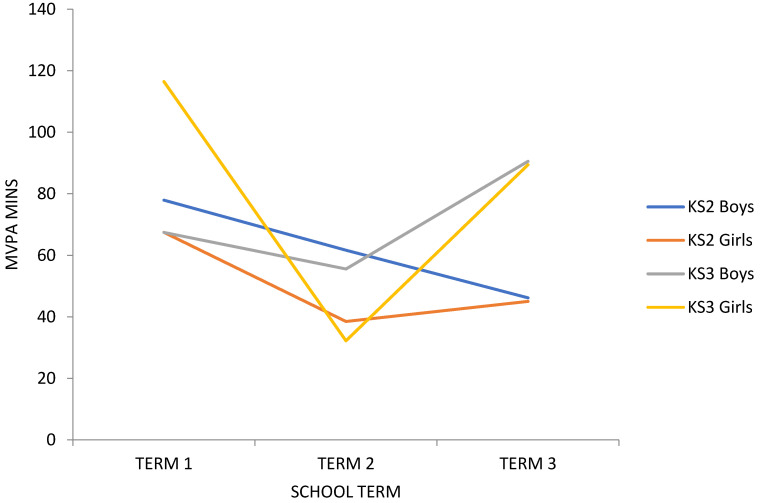
Mean daily moderate–vigorous physical activity (MVPA) time (mins) according to gender within key stage each school term.

Term 1 revealed the greatest number of children who met meet the 60 min PA guidelines [27], and term 3 revealed the fewest (term 1 = 47%, term 3 = 29%). However, there were gender, key stage (KS), and gender within key stage differences. All the groups except KS3 boys revealed the greatest number meeting the 60 min PA guidelines in term 1 (boys = 52%, girls = 41%, KS2 = 50%, KS3 = 42%, KS2 boys = 60%, KS2 girls = 36%, KS3 girls = 47%), whereas the results showed the greatest number of KS3 boys meeting the 60 min PA guidelines in term 2 (KS3 boys = 43%). Term 3 revealed the lowest number of boys, with the KS2 and KS2 boys meeting the 60 min PA guidelines (boys = 27%, KS2 = 26%, KS2 boys = 23%). Term 2 revealed the lowest number of girls, with the KS3 and KS3 girls meeting the 60 min PA guidelines (girls = 24%, KS3 = 25%, KS3 girls = 15%). The lowest number of KS2 girls meeting the 60 min PA guidelines was revealed in terms 2 and 3 (KS2 girls = 33%), and the lowest number of KS3 boys meeting the 60 min PA guidelines was revealed in terms 1 and 3 (KS3 boys = 33%). The= MVPA and meeting PA guideline descriptions are provided in Table 2, and patterns of MVPA across the school year are provided in Figure 1.

The rigour of the data collected was further tested, exploring children who provided data within the study. The objective of carrying out this analysis was to determine whether the children who provided data for term 1 only were those who engaged in less PA, and whether the children who provided data for two or three terms were the more active children. Therefore, the children were grouped into two categories based on whether they provided data for term 1 only, or whether they provided data for a minimum of two terms. 

The results showed that 16 children reported information for term 1 only, and 134 children provided information for a minimum of two terms. The MVPA levels appeared to be similar across the two groups, with the “term 1 only” group reporting a mean of 142.7 (±213.3) minutes MVPA compared with the “2 or more terms” group, reporting 132.5 (±189.9) minutes of MVPA. In addition to this, datasets from both groups of children were analysed to explore the numbers of children meeting the 1 h daily MVPA guidelines [28]. The “term 1 only” group reported 9/16 children (56%) as meeting the PA guidelines and 7/16 children (44%) not meeting the PA guidelines. The “2 or more terms” group reported 72/134 children (54%) as meeting the PA guidelines and 62/134 children (46%) as not meeting the PA guidelines. Therefore, it can be concluded that the children who provided data for term 1 only did not appear to be the least active children, and that children within the study appear to be consistently active across the three school terms.

### 3.2. Children’s MVPA within Different Environments across the School Year

When investigating HR according to location, the results revealed two statistically significant findings. The school MVPA over the year as a whole showed older children to engage in significantly greater MVPA at school compared with younger children (F₁₁,₁₃₅ = 4.948, *p* = 0.028, Eta^2^ = 0.035, KS2 = 20.2 min ± 27.1, KS3 = 34.3 min ± 52.5). Additionally, the motorised transport MVPA between school terms revealed statistically significant findings (F₁₁,₁₃₅ = 6.792, *p* = 0.002, Eta^2^ = 0.091). A Bonferroni pairwise comparison revealed a statistically significant finding between terms 1 and term 2 (motorised transport: term 1 MVPA = 8.1 min, term 2 MVPA = 1.1 min; *p* = 0.001), and between terms 1 and term 3 (motorised transport: term 1 MVPA = 8.1 min, term 3 MVPA = 1.9 min; *p* = 0.004). There were no significant findings when exploring MVPA according to home, on foot, outdoors, and other indoor locations (*p* > 0.05).

**Figure 2:**
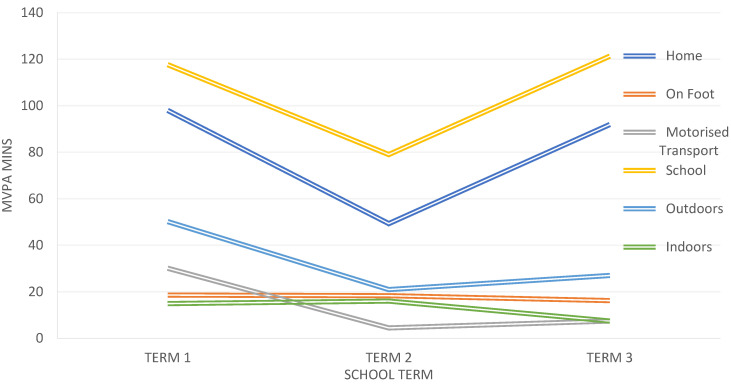
Mean daily moderate–vigorous physical activity (MVPA) time (mins) according location each school term.

**Figure 3:**
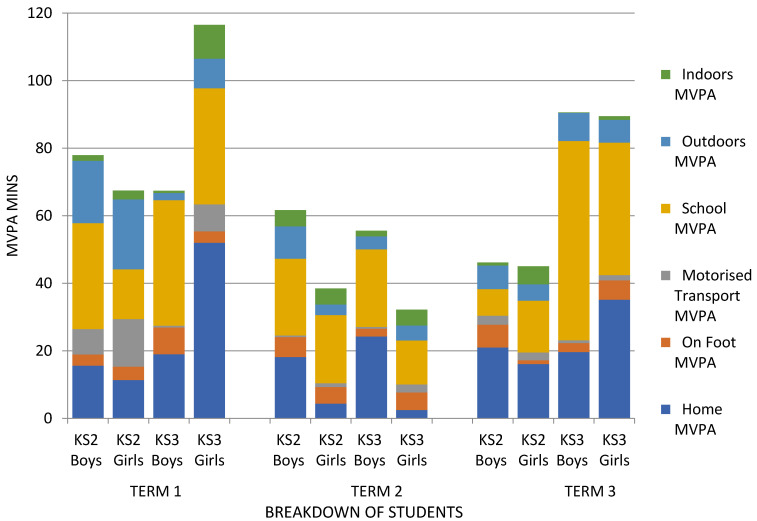
Mean daily moderate–vigorous physical activity (MVPA) time (mins) in each location according to gender within key stage each school term.

The patterns of MVPA according to location across the school year are illustrated in Figure 2, and patterns of MVPA according to gender within key stage per location across the school year are illustrated in Figure 3.

### 3.3. Children’s Perspectives on the Surrounidng Environment.

The Social–Ecological Model underpinned questions/topics which were discussed in the children’s focus groups. The focus groups allowed research questions to be explored further and results were produced regarding the locations visited according each school term. Boys and girls in both KS2 and KS3 discussed similar topics within their focus groups, and these were highlighted within the themes. The social–ecological model components [21] underpinned the first-order themes of the focus group transcripts. Following this, second-order themes highlighting barriers to PA or facilitators for PA were provided. The focus group themes and raw data are provided in Table 3. Additional comments from the data collection are provided below.

#### 3.3.1. Barriers to Physical Activity

##### “Organisational” Element

Children discussed restrictions around rules which may have been set by parents/guardians which limited the areas the children could access for PA: “…not being allowed to go outside or anything like, like being set rules like you can’t go very far or anything.” This meant that children were only able to access local areas, which may not all necessarily promote PA.

##### “Interpersonal” Element

Family commitments and responsibilities were highlighted as a potential barrier: “…if you have little sisters you have to look after, or little brothers and you sometimes struggle…” This highlights how children identify family responsibilities as a potential limitation to engaging in PA. 

In addition to this, the children discussed how peers can also influence PA behaviours: “Friends, say that you want to do something active and they just want to like sit around…” This indicated that sedentary behaviour amongst peers is likely to limit PA.

##### “Community/Policy” Element

The lack of a rewards system within the school regarding PA was highlighted as a barrier to PA, and the children felt that they could be rewarded for PA participation which in turn would encourage a greater number of children: “…you could like as rewards, like if you, you know at lunchtimes, you don’t have to do this but if you choose to like run like, every one hundred metres you run round the field, like if it’s obviously dry, you get like a reward or something.”

In addition to this, the children made reference to the types of activities which were staged, and particularly disliked how they were not consulted on the PA being offered: “There’s like a lot of people that don’t want to do the physical activity like the football and the rugby, and a lot of people like prisoner and dodgeball.”; “See what they like the most and start things to do with them.” This indicates that children recognise how their preferences of PA will help to encourage greater participation.

#### 3.3.2. Facilitators for Physical Activity

##### “Organisational” Element

The location of PA was highlighted as a reason influencing PA: “Because they’re your team and it’s the usual place to go and that’s where your team plays.” This indicates that the accessibility to PA locations influenced children’s motivation to take part in PA with peers. Similar findings were revealed with regards to family: “Not only is it helping my dad get prepared for the marathon, it’s also helping me with my legs so that I can run faster as well.”

##### “Interpersonal” Element

Children discussed the importance of the social environment which would encourage others to engage in PA: “…it’s got to be somewhere where they know other people because otherwise, they won’t go because they don’t know anyone else.” This highlights how children’s PA is encouraged when their peers are also present. The school would therefore be an ideal location, as it encompasses both peer groups and a common time where the children could engage in PA together. 

##### “Community/Policy” Element

The promotion of PA discussions showed child consultation as an important factor: “How about have a lunchtime fitness club, how about that?”; “Persuade them like how good sport is and everything so they can take part in sports.” The children also applied this to activities relating specifically to PE lessons: “Give them a choice of what they want to do for like P.E or something, so what they want to do for P.E.” This indicates that children would also like to have a degree of input when deciding on the activities involved in taught curricular PE lessons. 

##### “Other” Element

Children highlighted how weather conditions affect their PA behaviour: “If it’s sunny then I think more people like to go outside.” This indicated that a warmer season, with increased sun exposure, positively affected children’s decisions to engage in PA. 

Finally, the children highlighted how certain activities were offered on a seasonal basis, which meant that there would be fewer opportunities throughout the rest of the year to engage: “…rather than having football just at one point in the year, have it all round because some people prefer that.” This highlights the children’s frustration at not being able to access certain PA for sustained periods of time due to either the school’s policy, curriculum guidelines, seasonal conditions, staffing, etc. Therefore, the children raised ideas of staging preferred PA throughout the year to encourage PA.

## 4. Discussion

The findings from this study are in accordance with the current literature, which shows that PA behaviour patterns differ according to season [4]. Children have been reported to have reduced levels of PA during winter months, particularly in UK-based school children [4], and the MVPA findings in this study show that children in term 2 (winter/spring term) engaged in the least amount of MVPA minutes (*p* < 0.05; MVPA = 48.2 ± 39.6), which is in accordance with previous findings [4]. The focus group data support this, as the children highlighted the negative impact that winter dark nights and poor weather conditions had on PA: “…if it’s in the winter, some people don’t have motivation because it’s quite cold and dark, and if it’s muddy.”; “…the weather, because you can’t really run on a track, if the track is covered in puddles because you’ll probably like slip over.”

Previous literature has suggested that the levels of PA decline with age [29,30,31,32]. However, the current study findings revealed no significant difference in children’s MVPA levels between the first and last school term, or between the KS2 and KS3 participants. Many UK-based schools adopt a two-tier school system, which involves a KS2 to KS3 transition (i.e., the primary school to secondary school move). However, the school within this investigation was a middle school, and the children remained within this school during the KS2 to KS3 transition. The consistency in a middle school environment could explain the consistency in PA behaviours across all three school terms, which showed that on average, the older children were more active than the younger children, which is in contrast to previous research findings [29,33,34]. In addition to this, within focus groups, the KS2 children indicated that participation in PA outside of the school environment was subject to parent permission, and further engagement in PA was dependent on family/friend commitments and responsibilities: “…not being allowed to go outside or anything like, like being set rules like you can’t go very far or anything.”; “…sometimes some of the people might have other plans.” This highlights how younger children’s independent mobility and family commitments may have had a negative impact on PA participation within the current sample.

KS2 children’s focus groups also highlighted a need for younger children to be consulted in designing PA extra-curricular programmes, which offered greater variety: “They might not like it, so they might not want to do it.”; “See what they like the most and start things to do with them.”; “Get the school more active and more sporty and more sports clubs.”; “…and also offer more opportunities for clubs…” This indicates that the opportunities for PA within this particular school encourage PA for older children specifically, and there is a need to consider PA opportunities for younger children. However, the school-based PA findings do support the concept of the school being a supportive platform for PA promotion [35,36,37,38], and therefore schools are effective locations for staging PA interventions.

Each term showed a reduced percentage of children meeting PA guidelines which is in contrast to previous research [28,39], however this may explained by a reduced sample set available for analysis each school term. The literature has consistently reported that girls are less active than boys [32,33,40,41,42,43,44,45]; however, within this study, the girls engaged in greater amounts of MVPA than boys in term 1 (autumn term) and term 3 (summer term). This gender MVPA novel finding may be explained by the opportunities provided for girls’ PA at the participating school, which may encourage greater MVPA for girls compared with other schools nationally. Furthermore, the objective tools used to measure HR within this study provide an accurate measure of children’s HR, whereas previous research has relied on children’s self-reported data, which has been associated with an overestimation of PA [34,46]. Children referred to how the GPS and HR monitors were associated with monitoring personal HR and PA clubs, and identified how they could be used to promote PA: “…you should do running club or some club where you get active and that like the GPS thing.”

The most frequently visited locations reported from the GPS data within this study were: home, school, other indoor locations (excluding home and school), motorised transport, on foot, and outdoors. Previous spatial analysis research indicates that children engage with the home, school, daily commute, and local environments—i.e., green space and parks [47,48,49]—and the locations visited by children within this study support this. It was decided to also create a location called “time outside” which combined time on foot with time outdoors. This would produce a more reflective indication of children’s total time spent outside.

Time spent in motorised transport is typically associated with lower intensities of PA (due to the seated/sedentary aspect of this transport method); however, the current study found the children to have elevated HR when in motorised transport. It was established that the school policy allowed children to leave school immediately after extra-curricular after-school PA clubs, without the need to get changed. This resulted in the children finishing an extra-curricular activity with an elevated HR, and then making use of either public transport (i.e., local bus) or parental transport; therefore, the children were engaged in sedentary behaviours, and the HR would gradually return to resting HR during this recovery period whilst traveling. Future HR investigation including motorised transport may decide to consider other objective measures (such as accelerometers) or have a standardised “break” period prior to measuring HR to account for a lag in HR.

The location data for all groups of children showed reductions in MVPA from term 1 to term 2, before increases in MVPA in term 3. This is with an exception for time spent indoors, which showed a greater MVPA in term 2 (winter/spring months) and MVPA time on foot, which remained relatively consistent across all three terms. Children may have engaged in greater indoor MVPA during colder, darker UK winter months, and this is in accordance with the previous literature exploring the seasonality difference in children’s PA [4].

Throughout the academic year, children highlighted barriers to PA, with many being referred to consistently each term. The first barrier to be discussed was time for PA, which literature has acknowledged [50]. Within the current investigation, time was associated with parents/guardians’, and home responsibilities, which consequently affected children’s PA: “Not having the time to do it…”; “It might be affecting them by their home routine.” In addition to this, the timing of PA clubs was identified to affect PA participation. Some children felt that if PA clubs were offered at the same time, then they would have to choose one to attend, whereas ideally, they would have liked to engage in both types of PA.

After school clubs were identified as limiting attendance, because the children would not have parental permission to attend, potentially due to transport arrangements or the safety aspects of allowing children to walk home independently [51,52]: “…not being allowed to go outside or anything like, like being set rules like you can’t go very far or anything.” This suggests that children enjoyed having school-based PA opportunities such as lunchtime, as it was more accessible, and was not dependent on transport arrangements, extra parental permission, etc. These factors should be considered in PA intervention design in order to widen participation.

The policy aspects of PA within the school were highlighted by children. Many children felt that they should be consulted with regards to the types and variety of clubs offered, as opposed to decisions being made by staff: “There’s like a lot of people that don’t want to do the physical activity like the football and the rugby, and a lot of people like prisoner and dodgeball”; “How about have a lunchtime fitness club, how about that?” Additionally, the children felt that the greater promotion and awareness of the clubs would help encourage others to attend: “…posters for like games and stuff which we could get to do after school that will get you fit and healthy.”; “They might not have anyone to encourage them to do it.”; “Persuade them like how good sport is and everything so they can take part in sports.” The children also proposed that popular PA clubs were offered more frequently throughout the school year, as currently some PA clubs were offered for half a term, potentially due to deteriorating weather conditions and staff commitments: “…rather than having football just at one point in the year, have it all round because some people prefer that.” This supports findings from previous literature that proposes that extra-curricular promotion and participation that is shaped by the student voice would increase the motivation for PA [53], and therefore there is a need for schools to consult children in designing extra-curricular PA programmes.

Children felt that poor weather conditions at certain times of the year limited PA options and their motivation to participate in PA: “…the weather, because you can’t really run on a track, if the track is covered in puddles because you’ll probably like slip over.” Football matches and training sessions were often cancelled in the winter months, and children described their behaviour as “have to stay inside”. The children also discussed the impact poor weather conditions had on motivation for PA engagement. Wet weather conditions specifically were highlighted as a hindrance for PA motivation: “…if it’s raining, I don’t think people will have the motivation.” In contrast to this, the children described “sunny weather” as an encouraging factor that supported and motivated them to participate in PA: “If it’s sunny then I think more people like to go outside.” This supports previous literature related to PA seasonal variation [4,10], and this should be considered when designing extra-curricular PA programmes and PA-related interventions.

The children discussed a rewards policy, where as part of the school’s rewards system, children would be recognised and rewarded for attending a number of PA clubs: “…you could like as rewards, like if you, you know at lunchtimes, you don’t have to do this but if you choose to like run like, every one hundred metres you run round the field, like if it’s obviously dry, you get like a reward or something.”; “…the longer you run, and the longer the distance, you get a better reward or something like that.” Following these findings, it is proposed that the school rewards policy could be used to encompass children’s PA which would therefore further encourage greater PA participation.

In accordance with previous literature, the children discussed how their family and peers could influence their PA participation [54,55]. The children highlighted friends and family to encourage PA engagement, largely due to the social aspects of the activities, where children met friends or family and could have a “catch up”: “Friends because they’ve got the same life as what you do, so you can do the same thing.” However, the children also outlined how, if peers did not wish to attend a PA club, then this would influence others’ decisions to attend. A similar pattern was discussed regarding family, where family encouragement (brothers, sisters, or parents) would influence their PA behaviour. Previous studies suggest that a less supportive family towards PA reduces children’s PA participation [56]: “…sometimes some of the people might have other plans”; “…if you have little sisters you have to look after, or little brothers and you sometimes struggle…”; “Friends, say that you want to do something active and they just want to like sit around…” This highlights the influence both family and friends have on children’s decisions to participate in PA, and future PA research should consider this in PA intervention design.

As suggested by the previous literature, the children suggested that if a club was based at a particular location, then this would explain their reasons for visiting that environment. The facilities at such clubs would also justify why PA would take place at these venues: “Because they’re your team and it’s the usual place to go and that’s where your team plays.” Therefore, future research may wish to explore locations and proximity when designing PA programmes. As findings from this study reveal, children are more likely to engage in PA if it is easily accessible and is not associated with great cost or equipment needs. It is concluded that children would prefer to be involved in the design of PA programmes, and it is suggested that this will encourage greater participation.

The combination of HR and GPS monitors provided information of HR intensities in the different locations the children visited. These provided an indication of PA behaviour according to location over a four-day period, and adds to the current literature which has used GPS and HR monitors to explore children’s commuting patterns [8,52,57,58]. In addition to this, the range of ages included within the study (9–13 years) enabled for differences in PA behaviour according to key stage, specifically between KS2 and KS3. However, the novelty of the study lies within the use of a UK-based middle school, where both KS2 and KS3 children are taught within the same school environment, which allows the researcher to explore key stage differences. The findings of this can be compared with research in UK-based two-tier school systems (i.e., primary and secondary schools), where KS2 children are taught in primary schools and KS3 children are taught in secondary schools.

The mixed-methods approach to measuring and exploring PA behaviours implemented in this study provided a greater understanding of children’s reasons for PA. The children’s voices added another dimension to the research and provided children with an opportunity to discuss their reasons and thought processes behind PA participation and potential barriers. It also gave the children an opportunity to reflect upon their personal PA behaviours and indicate how they felt PA participation could be further supported. This highlights the importance of consulting children when designing school extra-curricular PA programmes, as children’s preferences of the types and timings of PA are likely to encourage greater levels of participation.

The repeated-measures design to this study allowed for information to be collected over each term across the school year, allowing for patterns of behaviour to be monitored over a greater period of time as opposed to one time point. This also allowed for investigation into whether children’s PA was affected by seasonality, as indicated by previous literature [4,10,59,60]. The children had the opportunity to voice their reasons for PA behaviour each school term, allowing for discussions to take place where the children could compare PA behaviours.

Previous literature raises concerns with the use of self-report measures, as these measures have been known to over-estimate the amounts of PA that have been completed, particularly when compared against more objective measures [61,62]. However, combining the use of these measures alongside GPS, HR monitors, and focus groups in a mixed-methods approach consequently supported the reliability of the produced data.

GPS data loss has been related to GPS device problems (e.g., signal failure, poor positioning, or low battery) and children’s handling—e.g., forgetting to turn on the device or forgetting to wear the device [63]. This investigation revealed reduced indoor GPS signal due to poor satellite connectivity. Therefore, to overcome this, the data were manually checked to confirm children were indoors based on their last GPS location point, i.e., child approaching an indoor location, before analysing HR data.

Finally, as previously discussed, the sample sizes for each term reduced when reporting HR and GPS data according to school term, with the final term including 41 children, compared with 58 children in term 1. This can be explained by the demands for participants to continue with the requirements of the investigation, and some children may have found it difficult to continue participating throughout the school year. Previous literature supports this, as it is suggested that sample size is not associated with data loss, but longer measurement periods are associated with greater data loss, and it seems that participant adherence decreases with time [63].

In accordance with the current literature, the findings from this study support the need for PA interventions to take into account the seasonal differences in children’s PA behaviours. The influence seasonal variation has on children’s PA may facilitate precise behaviour change interventions, which can be with greater intensity during periods of the year when PA levels are at their lowest [4]. The current literature confirms that there should be an increased focus on engaging youth in PA during unstructured leisure time [64]. Additional literature also highlights the importance of intrinsic motivation on PA engagement [65], and future research may wish to consider this in relation to PA seasonality. From the findings of this study, it is recommended that schools are an ideal platform to support children’s PA, particularly during the winter months associated with the winter/spring term. Break and lunchtime periods are perfectly placed to offer children an accessible opportunity to engage in PA and can attract a higher attendance rate. However, it is advised that children are consulted in the design of PA programmes to ensure that PAs offered are in line with children’s preferences.

## 5. Conclusions

This study provided an insight into children’s PA behaviour and PA location across the school year. Little research has implemented a repeated-measures mixed-method design across the school year incorporating the use of GPS technology. In support of previous research findings [4,64], the results from this study support how seasonal variation has been associated with differences in PA, with the winter/spring term comprising reduced daylight hours and poorer temperatures, resulting in significantly less PA.

Previous literature suggests that boys are more active than girls [28,39] and that there is an age-related decline in children’s PA [34,43,66]. However, this study’s findings contrast this, as during term 1 (Autumn term) and term 3 (Summer term), the girls engaged in greater MVPA than boys, and the KS3 children engaged in greater school-based MVPA across the school year compared with KS2 children.

The children highlighted the importance of being consulted in the design of school-based extra-curricular PA programmes, and indicated that poor weather conditions, technology, and family/peers were influential in PA participation. A PA programme that was well promoted, attached to a school reward system, and easily accessible was suggested to encourage greater participation. The study extended PA knowledge exploring gender and age interactions in a school-based sample, and highlights how the Social–Ecological Model can be used to underpin a mixed-methods research design.

## Figures and Tables

**Table 1:** Breakdown of the children each school term according to the school key stage (key stage 2 = 9–11 years; key stage 3 = 11–13 years) and gender.

Key Stage (and Age Range)	Term 1 (Autumn Term)			Term 2 (Winter-Spring Term)			Term 3 (Summer Term)		
	Boys	Girls	Total	Boys	Girls	Total	Boys	Girls	Total
2 (School Years 5–6, 9–11 years of age)	20	14	34	18	12	30	13	6	19
3 (School Years 7–8, 11–13 years of age)	9	15	24	7	13	20	9	13	22
Total	29	29	58	25	25	50	22	19	41

**Table 2:** Mean daily moderate–vigorous physical activity (MVPA) time, and the number of children meeting the physical activity (PA) guidelines according to each school term.

	Term 1 (Autumn Term)			Term 2 (Winter/Spring Term)			Term 3 (Summer Term)		
Variable	MVPA (min)	Meeting PA Guidelines		MVPA (min)	Meeting PA Guidelines		MVPA (min)	Meeting PA Guidelines	
		N	Percent (%)		N	Percent (%)		N	Percent (%)
Boys	74.7 (±58.1)	15	52%	60 (±44)	12	48%	64.3 (±73.8)	6	27%
Girls	92.9 (±98.5)	12	41%	35.5 (±30.1)	6	24%	75.4 (±85.2)	6	32%
KS2 (9–11 years)	73.6 (±59.6)	17	50%	52.4 (±42.2)	13	43%	45.8 (±46.4)	5	26%
KS3 (11–13 years)	98.1 (±103.3)	10	42%	41.3 (±34.9)	5	25%	89.9 (±94.5)	7	32%
KS2 Boys	77.9 (±57.2)	12	60%	61.7 (±46.5)	9	50%	46.2 (±51.2)	3	23%
KS2 Girls	67.5 (±64.4)	5	36%	38.5 (±31.4)	4	33%	45 (±38)	2	33%
KS3 Boys	67.4 (±62.8)	3	33%	55.6 (±39.8)	3	43%	90.6 (±95.1)	3	33%
KS3 Girls	116.6 (±119.6)	7	47%	32.2 (±29.8)	2	15%	89.4 (±98)	4	31%
Overall	83.8 (±80.6)	27	47%	48.2 (±39.6) *	18	36%	69.5 (±78.4)	12	29%

* Statistically significant difference between school term, gender, key stage, and gender within key stage (*p* < 0.05). MVPA = Moderate-vigorous physical activity.

**Table 3:** A summary of themes and examples of raw data extracts for barriers to and facilitators for PA.

Themes	Selected Quotes From Children	
	Barriers to PA	Facilitators for PA
Individual Time	“If I have too much homework, exams and stuff.” (Term 1—Autumn) “It might be affecting them by their home routine.” (Term 2—Winter/Spring term) “Not having the time to do it…” (Term 3—Summer term)	“…however long you do on a physical activity, you get half that time on technology or something like that” (Term 1—Autumn) “Like having a certain time or place where you can do a P.E challenge in school time” (Term 2—Winter/Spring term) “Make it more often because we only have two lessons of it (Physical Education), we should have more.” (Term 3—Summer term)
Interpersonal Friends and family	“Might have to look after their family and it might stops [sic] you having their social time with their friends and going out” (Term 1—Autumn) “…say there’s something wrong going on in the family and they’ve got to go and help, it’s fitting into their routine.” (Term 2—Winter/Spring term) “…if you have little sisters you have to look after, or little brothers and you sometimes struggle…” (Term 3—Summer term)	“I do runs with my dad, I go on my bike” (Term 1—Autumn) “Not only is it helping my dad get prepared for the marathon, it’s also helping me with my legs so that I can run faster as well.” (Term 2—Winter/Spring term) “Friends because they’ve got the same life as what you do, so you can do the same thing.” (Term 3—Summer term)
Organisational Location/Environment	“…a club that’s fun and active, and people would like to go to it and it’s not too far.” (Term 1—Autumn) “…not being allowed to go outside or anything like, like being set rules like you can’t go very far or anything.” (Term 2—Winter/Spring term) “The availability of places that you go to do it.” (Term 3—Summer term)	“I go up to the college car park because it’s big and loads of my friends just play there.” (Term 1—Autumn) “Because they’re your team and it’s the usual place to go and that’s where your team plays.” (Term 2—Winter/Spring term) “It helps to be somewhere local to most people…” (Term 3—Summer term)
Community/Policy Child Voice	“People might not like what the variety is” (Term 1-Autumn) “There’s like a lot of people that don’t want to do the physical activity like the football and the rugby, and a lot of people like prisoner and dodgeball” (Term 2—Winter/Spring term) “See what they like the most and start things to do with them.” (Term 3—Summer term)	“You can ask what they like the most, and start clubs and get them to come.” (Term 1—Autumn) “How about have a lunchtime fitness club, how about that?” (Term 2—Winter/Spring term) “Give them a choice of what they want to do for like P.E or something, so what they want to do for P.E.” (Term 3—Summer term)
Other Weather	“…if it’s in the winter, some people don’t have motivation because it’s quite cold and dark, and if it’s muddy.” (Term 1—Autumn) “…if it’s raining, I don’t think people will have the motivation.” (Term 2—Winter/Spring term) “…the weather, because you can’t really run on a track, if the track is covered in puddles because you’ll probably like slip over.” (Term 3—Summer term)	“…you need the activity, the exercise and the nice fresh air, instead of being stuck indoors, stuff like that.” (Term 1—Autumn) “If it’s sunny then I think more people like to go outside.” (Term 2—Winter/Spring term) “…rather than having football just at one point in the year, have it all round because some people prefer that.” (Term 3—Summer term)
